# Synthesis, Characterization and Antioxidant Properties of a New Lipophilic Derivative of Edaravone

**DOI:** 10.3390/antiox8080258

**Published:** 2019-07-31

**Authors:** Cristina Minnelli, Emiliano Laudadio, Roberta Galeazzi, Dario Rusciano, Tatiana Armeni, Pierluigi Stipa, Mattia Cantarini, Giovanna Mobbili

**Affiliations:** 1 Dipartimento di Scienze della Vita e dell’Ambiente (DISVA), Università Politecnica delle Marche, via Brecce Bianche, 60131 Ancona, Italy; 2 Dipartimento S.I.M.A.U., Università Politecnica delle Marche, via Brecce Bianche, 60131 Ancona, Italy; 3 Sooft Italia SpA, Contrada Molino 17, 63833 Montegiorgio, Italy; 4 Dipartimento Scienze Cliniche Specialistiche ed Odontostomatologiche, Università Politecnica delle Marche, via Brecce Bianche, 60131 Ancona, Italy

**Keywords:** antioxidants, edaravone, liposomes

## Abstract

As part of a program aimed to obtain antioxidants able to interact with cell membrane, edaravone (EdV, 3-methyl-1-phenyl-2-pyrazolin-5-one), a well-known free radical scavenger, has been modified by alkylation at its allylic position (4) with a C-18 hydrocarbon chain, and the increased lipophilicity has been determined towards the interaction with liposomes. The obtained derivative has been studied by means of density functional theory (DFT) methods in order to characterize its lowest energy conformers and predict its antioxidant properties with respect to the parent compound EdV. The in vitro antioxidant activity of C18-edaravone was studied by means of the α,α-diphenyl-β-picrylhydrazyl (DPPH) assay and in lipid peroxidation experiments performed on artificial lipid membranes using water-soluble as well as lipid-soluble radical initiators. Moreover, since oxidative stress is involved in numerous retinal degenerative diseases, the ability of C18-edaravone to contrast 2,2-azobis (2-amidinopropane hydrochloride) (AAPH)-induced cell death was assessed in adult retinal pigmented epithelium (ARPE-19) cells. Overall, the results demonstrated that the newly synthesized molecule has a high affinity for lipid membrane, increasing the efficacy of the unmodified edaravone under stress conditions.

## 1. Introduction

**Figure 1:**
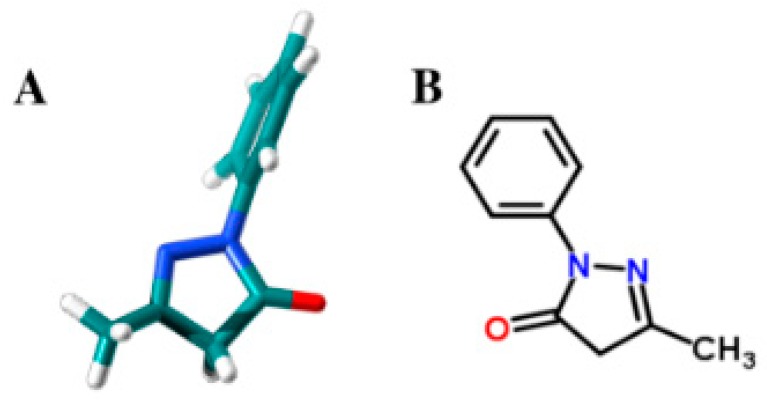
Edaravone 3D structure (**A**) and 2D structure (**B**).

Pyrazolones are synthetic oxo-derivatives of five-membered unsaturated heterocycles with two adjacent nitrogen atoms. They are usefully employed in many fields and, in particular, as pharmaceuticals, they exhibit bioactivity as an analgesic, antibacterial, anti-inflammatory, antioxidant and antitumoral [1,2,3]. Among pyrazolones, edaravone (EdV, 3-methyl-1-phenyl-2-pyrazolin-5-one) (Figure 1) represents the first free radical scavenger clinically approved in Japan since 2001 as neuroprotective agent [4,5].

**Scheme 1:**
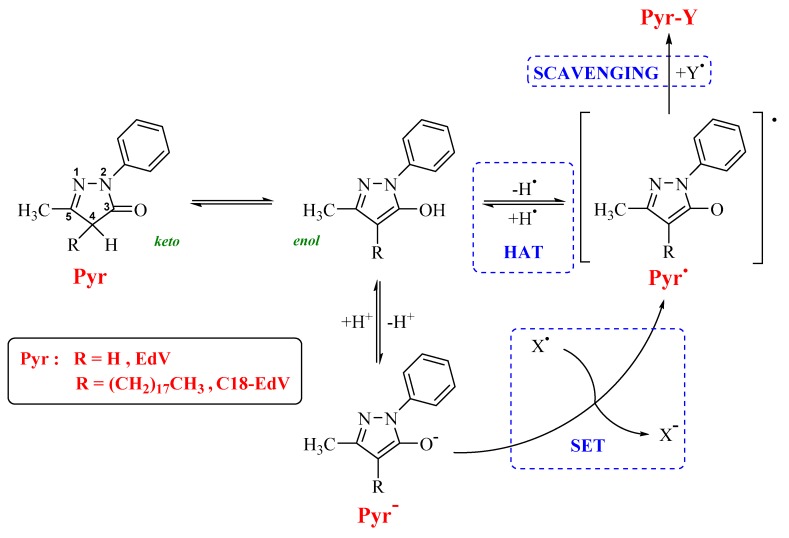
Pyrazolone (Pyr) antioxidants, EdV and C18-EdV, showing the ring atoms numbering and the antioxidant mechanisms accepted [7].

This molecule was discovered in the course of a research program aimed at developing antioxidants for the treatment of acute cerebral infarction, characterized by a phenol-like free radical-scavenging activity (H-donors antioxidants). The purpose was to synthesize an aromatic heterocyclic structure where a hydroxyl group would be generated by keto-enol tautomerization to obtain the key functional group responsible for the radical-scavenging activity as in phenol derivatives. A number of heterocyclic rings containing an amide or a ketone moiety were synthesized and edaravone, a weak acid with a dissociation constant (pKa) of 7.0, was identified as an active compound. Therefore, in a physiological environment (pH, 7.4), the percentages of the neutral and anionic forms are about 29% and 71%, respectively [6]. In addition, the neutral form of the EdV undergoes a marked keto-enol tautomerism that, together with the presence of the anionic form, constitutes the basis of its antioxidant activity. The antioxidant activity of these derivatives can be attributed to its possibility to undergo single electron transfer (SET) processes and hydrogen atom transfer (HAT) reactions, both yielding species with an unpaired electron delocalized in the heterocyclic ring, while the radical scavenging activity lead in general to non-radical products (Scheme 1).

This synthetic antioxidant molecule is able to quench hydroxyl, peroxyl and superoxide radicals and showed antioxidant activity against lipid peroxidative damage induced by water- or lipid-soluble radicals [7,8]. These properties make edaravone as a promising candidate for the treatment of diseases associated with oxidative stress. Since the eye contains one of the highest oxygen-consuming tissue in the human body, many ocular disorders are linked with oxidative stress, including age-related macular degeneration (AMD), glaucoma, diabetic retinopathy (DR), retinal vein occlusion (RVO) [9] and dry eye disease [10]. Moreover, the high quantity of polyunsaturated fatty acids (PUFAs) in the optical disk membranes, photoreceptors and retinal synapses, [11] makes the retinal lipids particularly susceptible to oxidative damage (lipid peroxidation, LPO). This process results in the alteration of membrane fluidity and permeability, in the inactivation of membrane-bound enzymes and receptors, and in a general loss of the membrane functionality. Membrane-targeting antioxidants could counteract or prevent the LPO; [12] there is, therefore, an increasing interest toward strategies aiming to potentiate the interaction between antioxidants and lipid systems [6,13,14,15]. In order to obtain an EdV analogue possessing these characteristics, in this study we synthesized a lipophilic derivative, alkylated at position (4) of the pyrazolone ring with a C-18 hydrocarbon chain. The in vitro activity of the resulting derivative, namely C18-EdV, was studied by DPPH assay and in lipid peroxidation experiments performed on artificial lipid membranes, by using water-soluble as well as lipid-soluble radical initiators. Moreover, a DFT study allowed us to individuate its lowest energy conformers and to predict its antioxidant properties. With the aim to investigate if the functionalization strategy increases the edaravone affinity with lipid bilayers, the interaction with liposomes was studied. Finally, after cytotoxicity evaluation of the synthesized derivatives, the ability of C18-EdV to contrast AAPH-induced cell death was assessed in adult retinal pigmented epithelium (ARPE-19) cells.

## 2. Materials and Methods

### 2.1. Materials

Commercial phosphatidylcholine from egg yolk (PC), edaravone, α,α-diphenyl-β-picrylhydrazyl (DPPH) and 2,2-azobis (2-amidinopropane hydrochloride) (AAPH) were obtained from Sigma Aldrich Co. (Stenheim, Germany). The 2,2-azobis(2,4-dimethylvaleronitrile) (AMVN) was purchased from Santa Cruz Biotechnology, Inc. (Dallas, USA) and used after purification by recrystallisation in cold methanol. All the other reagents and chemicals were of analytical grade for biochemical purposes or HPLC grade. All the solutions were prepared in ultrapure MilliQ water to prevent metal contamination. The materials and reagents employed in the synthetic procedures, performed in an argon atmosphere, were purchased from Sigma Aldrich Co. (Stenheim, Germany) and used without purification. All solvents were analytically pure and dried before use. TLC was carried out on aluminum sheets precoated with silica gel 60 F254 (Merck). Column chromatography was performed using silica gel 60 (230–400 mesh). Mass spectra (MS) were recorded on a Hewlett-Packard spectrometer 5890, series II, in electron impact mode. The ^1^H and ^13^C NMR spectra were recorded at 400 and 100 MHz, respectively, on a Varian Gemini 400 spectrometer, using CDCl_3_ or DMSO as solvents. Chemical shifts (δ) are reported in ppm relative to TMS and coupling constants (J) in Hz. Adult human retinal pigment epithelial (ARPE-19) cells were provided from Fidia-Sooft. All cell culture reagents were purchased from Euroclone (Euroclone, Italy). ARPE 19 CELLS (CRL 2302) were obtained from American Type Culture Collection (ATCC) Manassas, Virginia, USA.

### 2.2. Synthetic Procedure

#### 2.2.1. Ethyl 3-Oxo-2-Octadecyl-Butanoate (Ethyl 2-Octadecyl-Acetoacetate), 2

To a stirred solution of sodium ethoxide in ethanol (21% wt, 22 mmol), ethyl acetoacetate 1 (2.53 mL, 20 mmol) was added at room temperature. The mixture was heated at 80 °C and after 30 min octadecyl bromide (6.83 mL, 20 mmol) in THF (4 mL) was slowly added over 2 h. After refluxing for 12 h, the mixture was cooled and acidified with HCl 1 M with vigorous stirring until neutral pH. Then, the mixture was extracted with ethyl acetate (3 × 10 mL). The organic layers were collected, combined and washed with brine (2 × 10 mL), dried over anhydrous Na_2_SO_4_ and concentrated. The residue was purified by silica gel column chromatography (cyclohexane: ethyl acetate 95:5) to give the desired product 2 (5.56 g, 72.7%). ^1^H NMR (CDCl_3,_ 400 MHz): δ 0.86 (t, J = 6.4 Hz, 3H), 1.16–1.33 (m, 32H + 3H, 7.2 Hz), 1.76–1.88 (m, 2H), 2.20 (s, 3H), 3.37 (t, J = 7.2 Hz, 1H), 4.17 (q, J = 7.2 Hz, 2H). ^13^C NMR (CDCl_3_, 100 MHz): 14.1, 22.7, 27.4, 28.2, 28.7, 29.3, 29.5, 29.6, 29.7, 31.9, 59.9, 61.2, 169.9, 203.4.

#### 2.2.2. 3-Methyl-4-Octadecyil-1-Phenyl-1H-Pirazol-5(4H)-One (C18-Edaravone)

Ethyl 3-oxo-2-octadecyl-butanoate (2 mmol) was added to phenyl hydrazine (2 mmol) under argon. The mixture was then heated at 140 °C and cooled in an ice-water bath after 5 h. A portion of 3 mL of diethyl ether was added to precipitate pyrazolone. The product was filtered and washed thoroughly with diethyl ether to obtain a white solid (72%). ^1^H NMR (DMSO, 400 MHz): δ 0.85 (t, J = 7.2 Hz, 3H), 1.17–1.31 (m, 32 H), 1.37–1.44 (m, 2H), 2.10 (bs, 3H), 7.12–7.19 (m, 1H), 7.41 (t, J = 8.0 Hz, 2H), 7.68–7.75 (m, 2H), 10.47 (bs, 1H, NH). ^1^H NMR (CDCl_3_, 400 MHz): δ 0.87 (t, J = 6.8 Hz, 3H), 1.14–1.37 (m, 32 H), 1.78–1.90 (m, 1 H), 1.94–2.05 (m, 1 H), 2.15 (s,3H), 3.26 (t, J = 5.6 Hz, 2H), 7.17 (t, J = 7.2 Hz, 1H), 7.39 (t, J = 8.1 Hz, 2H), 7.89 (d, J = 8.1 Hz, 2H), ^13^C NMR (CDCl_3_, 100 MHz): δ 14.1, 15.7, 22.7, 25.4, 27.5, 29.2, 29.3, 29.5, 29.6, 29.6, 29.7, 31.9, 52.4, 118.7, 124.8, 128.8, 138.1, 160.0, 173.4. ESI-MS: (m/z.) 424.7 [M-H]-. IR (KBr, cm^−1^): 2918, 2851, 1604, 1555, 1501, 1471, 1392, 1314. Combustion elemental analysis calculated for C_28_H_46_N_2_O: C, 78.82; H, 10.87; N, 6.57. Found: C, 78.86; H, 10.84; N, 6.61. 

### 2.3. Computational Methods

Gaussian 09 suite of programs has been used for electronic calculations, [16] using the B3LYP functional [17] and the 6-311++G (d, p) basis set, the PCM continuum model (water) was used for solvent effect prediction [18]. This functional reliability has been extensively proven [19,20,21,22]. The prediction of the C18-EdV antioxidant activity derivative has been related to that of EdV using the relative values of energy (ΔE_iso_), and bond dissociation energy (BDE). All compounds initially underwent a complete conformational search using the MC/MM method and AMBER force field as implemented in the Schrödinger Suite 2014 [23]. The lowest energy conformers were subsequently optimized at DFT level without any symmetry constraints and were confirmed to be real minima by frequency calculation (no imaginary frequency). The bond dissociation energies (BDE) of the C–H bond in Pyrazolone position 4 and its formation were calculated as the energy difference between the neutral molecule (Pyr) and the corresponding radical (Pyr•) plus hydrogen (H) (Equation (1)).

BDE_CH_ = [E(Pyr•) + E(H)] – E(Pyr). (1)

The radical stability has been determined by the calculation of stabilization energies (ΔE_iso_) according to literature reports [24].

### 2.4. DPPH Assay

The DPPH assay was performed according to the following procedure. Appropriate aliquots of antioxidant solutions in methanol were mixed with a 100 µm DPPH methanolic solution (antioxidant 25 µM, DPPH 100 µM, FC); the reaction mixtures were shaken vigorously and incubated for 30 min in the dark. The absorbance at 517 nm was then determined with a spectrophotometer (BioTek Synergy HT MicroPlate Reader Spectrophotometer) against a blank which lacked in DPPH. The percent inhibition of the DPPH radical by antioxidants was calculated according to the following equation:

Inhibition ratio (%) = [(A_control_-A_sample_)/A_control_] × 100

where A_control_ is the absorbance of the control obtained by adding to DPPH methanol solution a methanol aliquot equal to the antioxidant solution volume added. A_sample_ is the absorbance of the reaction solution at 30 min. Each sample was measured in triplicate. Mean and standard deviation (*n* = 3) were calculated.

### 2.5. Liposome Preparation

Lipid peroxidation inhibition was carried on egg yolk PC large unilamellar or multilamellar liposomes as membrane models. Unilamellar liposomes were also employed in the interaction studies of antioxidants with liposome cell membrane models. All calculations were made by considering an average molecular weight of egg yolk PC of 768 u.m.a. 

#### 2.5.1. Multilamellar Vesicles (MLVs)

Liposomes were prepared by the “thin film hydration” method. Chloroform stock solutions (10 mm) were prepared for each tested compound. Appropriate amounts of chloroform solutions of L-α-phosphatidylcholine (100 mg·mL^−1^) were taken up in a round bottom flask; the tested antioxidant compound (10 mm in CHCl_3_) was added in a 1:50 molar ratios to the lipid. When required, appropriate aliquots of stock solutions in chloroform of the lipophilic radical initiator AMVN were added. The solvent was slowly evaporated with a stream of nitrogen and the thin film obtained was dried for at least 2 h under reduced pressure. An appropriate amount of 20 mm phosphate-buffered saline solution (pH 7.4) was added and the film was peeled off by vortexing to obtain a milky aqueous suspension (3 mm PC, 0.06 mm antioxidant, 1 mm AMVN FC). The multilamellar liposomes were incubated overnight to swell and stabilize.

#### 2.5.2. Large Unilamellar Vesicles (LUVs)

Unilamellar liposomes (LUVs) were prepared by extrusion of MLV suspensions by passing through two stacked polycarbonate filters (Whatman, Nucleopore, pore size 200 nm) using the Mini Extruder from Avantipolar Lipids Inc. (Alabaster, AL, USA). After 21 extrusion steps, a homogeneous population of LUV was obtained, as assessed by dynamic light scattering [25,26]. The liposome suspensions were used in the AAPH-peroxidation studies. PC Large unilamellar vesicles were also employed in the interaction studies of antioxidant with liposome cell membrane models.

### 2.6. Peroxidation of PC Liposomes

Lipid peroxidation was induced either within or outside the bilayer of egg yolk PC liposomes by peroxyl radicals, generated at a constant rate by thermal degradation of lipophilic or hydrophilic azocompounds [27]. Control experiments were carried out without antioxidant addition. The procedure used varied slightly depending on the type of initiator. When AAPH was used as the water-soluble one, 300 µL portions of each LUV dispersion with and without the antioxidant were taken up and added with 25 µL AAPH 65 mm (5 mm FC) and incubated for 2 h at 37 °C, whereas another 300 µL was added with 25 µL of PBS, 10 µL of 10 mm ethanolic BHT to be used as controls (non-oxidized samples). When instead the lipid-soluble initiator (AMVN) was used, 600 µL of each MLV suspension (with and without antioxidant) containing AMVN 1 mm and prepared as described above, was incubated at 56 °C for 2 h, whereas other 600 µL were added with 10 µL of 10 mm ethanolic BHT to be used as controls (non-oxidized samples). After the incubation time, 10 µL of 10 mm ethanolic BHT was added to stop the reaction and to prevent possible peroxidation of PC during the thiobarbituric acid reactive substance (TBARS) assay. The samples were added to 900 µL of TBA–TCA–HCl (0.375% w/v TBA, 15% w/v TCA, 0.2 M HCl) and heated for 15 min at 95 °C. The test tubes were then cooled and centrifugated at 5000 rpm for 10 min. The determination of the aldehydic breakdown products of lipid peroxidation (TBARS) was made in the supernatant by measuring at 532 nm the absorbance of the pink chromophore developed upon heating. The antioxidant activity of the C18-edaravone and edaravone was expressed as % inhibition according to the following equation:

% inhibition = (1 − ΔA_sample_/ΔA_PC_) × 100

where ΔA_sample_ is the difference of the absorbance between the oxidized and non-oxidized sample containing the antioxidants and ΔA_PC_ is the difference of the absorbance between the oxidized and non-oxidized PC. All the experiments were repeated at least three times and measurements were run in triplicate.

### 2.7. Studies on Interaction of Antioxidants with Liposome Cell Membrane Models

In typical incorporation experiments, different concentrations of edaravone and C18-edaravone in 0.1 mL of methanol were added to 0.9 mL of PC LUV in PBS (3 mm, lipid final concentration; 0.25, 0.5, and 1 mm antioxidant final concentration). The liposomal emulsions were vortexed and incubated for 20 min at 37 °C. Next, the EdV and C18-EdV were separated from liposomes containing the molecules by size exclusion chromatography. The hydrated Sephadex G-50 resin was placed into disposable syringes (2.5 mL) inside 15 mL plastic test tubes and then preconditioned with PBS. After having gently added 1 mL of EdV or C18-EdV liposomal suspensions on the top of the syringes, the samples were centrifuged at 500 g for 10 min. Free EdV or C18-EdV were also used as controls. The eluates were collected at the bottom of the test tubes and the Stewart assay was carried out to determine the lipid content in the liposome preparation after gel filtration. On hundred and fifty microliter samples of liposomes separated from the unentrapped antioxidant. The amount of antioxidants interacting with the liposome membrane was evaluated after liposome lysis obtained by addition of Triton X-100 (FC 1% v/v). The molecule concentrations were estimated by the Folin-Ciocalteu assay [28]. 150 μL of 50% Folin-Ciocalteu reagent was added to 50 μL of every sample into a 96-well microplate and shaken. After 10 min 0.100 mL of a 7.5% sodium carbonate solution was added, the mixtures were incubated in the dark for 1 h at room temperature and the absorbance measured at 765 nm on a BioTek Synergy HT MicroPlate Reader Spectrophotometer using an appropriate blank. Calibration curves were plotted for both edaravone and C18-edaravone. The ratio (%) of EdV and C18-EdV incorporated into the lipid bilayer at a certain concentration was calculated as follow:

% ratio of incorporated antioxidant = 100 × [incorporated amount]/[added amount].

All the experiments were repeated at least three times and measurements were run in triplicate.

### 2.8. Cell Treatment

ARPE19 cells were maintained in 25 cm^2^ flasks in complete DMEM/F12 medium at 37 °C, 5% CO_2_ and 95% relative humidity. Complete DMEM/F12 medium was prepared by adding 10% (v/v) heat-inactivated fetal bovine serum (FBS), 2 mm glutamine and 100 U/mL penicillin-streptomycin. Culture medium was changed every 2 days until cells grew to 90% confluence. The cell cultures were detached by trypsinization with 0.5% trypsin in PBS containing 0.025% EDTA and counted using trypan blue exclusion assay. 

For treatments, ARPE19 cells were seeded in 24-well plates at 8 × 10^4^/well to reach 50%–60% of confluence at 24 h. In the cytotoxicity assay, the cells were incubated for 24 h with increasing concentrations (40–320 μm) of EdV and C18-EdV. For the cytoprotection assay, edaravone and C18-edaravone were tested in the 40–240 μm concentration range. After the time of incubation, the cells were washed twice with PBS and treated with AAPH (FC 10 mm) for 24 h. Previous MTT viability assays for cytotoxicity studies were performed in order to establish the combination of dose/time of AAPH treatment (data not shown).

### 2.9. Assay of Mitochondrial Viability (MTT Assay)

The mitochondrial activity of the cells and therefore the cell viability was evaluated by using 3-(4,5-dimethylthiazol-2-yl)-2,5-diphenyltetrazolium bromide (MTT) assay [29]. At the time of the analyses, the media was discarded from each well and replaced with fresh medium supplemented with MTT at a final concentration of 100 µg·mL^−1^. ARPE19 cells were incubated for 3 h at 37 °C in 5% CO_2_ atmosphere and 400 μL of DMSO were then added to each well and mixed thoroughly by shaking to solubilize the purple formazan crystals formed from MTT reduction after incubation. The absorbance was read on a multiwell scanning microplate reader (BioTek Synergy HT MicroPlate Reader Spectrophotometer) at 570 nm using the DMSO as blank. The optical density in the control group (untreated cells) was considered as 100% viability. The relative cell viability (%) was calculated as follow:

Cell viability (%) = (A570 of treated samples/A570 of untreated samples) × 100.

Each experiment was performed at least five times in triplicate. 

### 2.10. Statistical Analysis

Data are presented as mean ± S.D. In the cellular experiments, statistical comparison of differences among groups of data was carried out using one-way analysis of variance (ANOVA), followed by Tukey’s post hoc test using GraphPad Prism. Values of *p* < 0.05 were considered statistically significant.

## 3. Results and Discussion

### 3.1. Synthesis and Characterization of C18-EdV

**Scheme 2:**
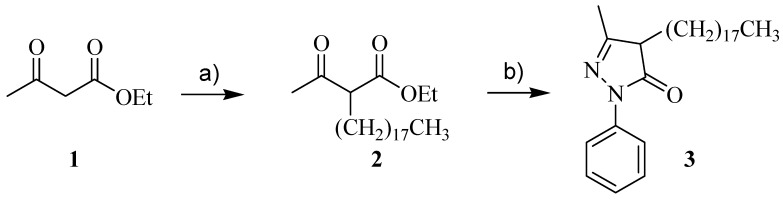
Reagents and conditions: (**a**) NaOEt, EtOH, r.t, then octadecylbromide in THF, 80 °C; (**b**) phenylhydrazine, CH_3_COOH, 120 °C.

The 3-methyl-4-octadecyl-1-phenyl-1H-pyrazol-5(4H)-one (C18-EdV) studied in the present work was synthesized according to the pathway reported in Scheme 2 using octadecyl bromide as alkylating agent. The condensation reaction of the alkylation product with phenylhydrazine allowed to obtain C18-EdV. 

The structure of the synthesized molecule has been confirmed by mass spectrometry (MS), infrared spectroscopy (IR), elemental analysis and nuclear magnetic resonance spectroscopy (NMR). ^1^HNMR spectrum of C18-EdV, carried out in CDCl_3_, reveals a triplet signal at δ 3.26 ppm, attributable to the 4-CH methyne proton. The ^1^HNMR spectrum registered in DMSO highlighted the presence of a different tautomeric form. The signal corresponding to the 4-CH methyne proton present in the ketonic form of C18-EdV disappears in the spectrum registered in DMSO where the stabilized form presents a strongly deshielded proton at about 10.5 ppm that could be assigned to an NH proton of the aminic form, in analogy to what reported for EdV [30]. These results indicate that the functionalization of edaravone with a long carbon chain does not prevent the tautomerism phenomenon in EdV-C18, which seems to represent one of the important prerequisites of pyrazolones antioxidant activity.

### 3.2. Scavenging Activity of DPPH Free Radical

**Figure 2:**
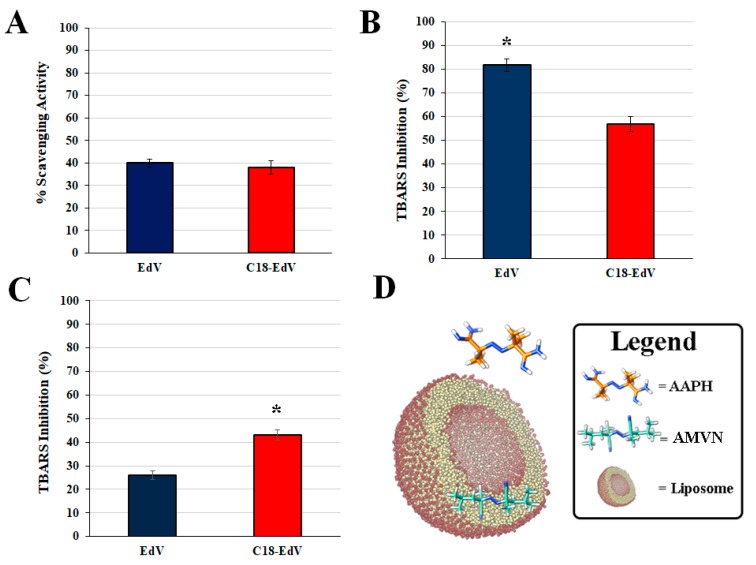
Antioxidant activity of edaravone (EdV) and C18-edaravone (C18-EdV) determined by means of different assays. DPPH free radical scavenging activity (**A**); percentage (%) of inhibition by Thiobarbituric acid reactive substances (TBARS) formation in phosphatidylcholine (PC) (3 mm) liposomes peroxidation by EdV or C18-EdV (0.06 mm) induced by 2,2-azobis (2-amidinopropane hydrochloride) (AAPH) (5 mm) (**B**) or 2,2-azobis(2,4-dimethylvaleronitrile) AMVN (1 mm) (**C**); schematic representation of water-soluble AAPH and lipid-soluble AMVN localization in a PC-liposome (**D**). Results are expressed as the mean ± standard deviation (*n* = 3). * *p* < 0.001.

The DPPH (2,2-diphenyl-1-picrylhydrazyl) assay was used as the first experimental approach to compare the radical scavenging activity of C18-EdV with that of the unmodified molecule EdV; the results are presented in Figure 2A as the percentage inhibition of DPPH radical scavenging activity.

As shown in the histogram, the C18-EdV retains the same scavenging activity of the parent compound and this means that the structural modification made did not influence the antioxidant power of the molecule. At this purpose, DFT studies were carried out to provide insight into EdV and C18-EdV structure-activity relationships.

### 3.3. Computational Results

In general, the antioxidant activity of these derivatives mainly involves the hydrogen atom transfer (HAT) and the single electron transfer (SET) mechanisms. In the HAT mechanism, the efficiency of an antioxidant is related to its ability to transfer an H atom to an incoming free radical, leading to the termination of further chain reactions (chain-breaking antioxidants) [31]. The ability to donate a hydrogen atom is mainly governed by the homolytic bond dissociation enthalpy (BDH). The gas-phase BDH_CH_ for EdV analogues, referring to the C-H bond at pyrazolone position 4, has been determined in previous theoretical studies [24,32,33], showing a good agreement with the experimental results. As a consequence, the antioxidant activity prediction of C18-EdV based upon this assumption was theoretically investigated at the DFT/B3LYP level of theory. In addition, the nature of the electron-donating group’s presence (EDGs) at position 4 is of considerable importance in the BDH_CH_, also in term of resonance stabilization with respect to the pyrazolonic radical produced. For this reason, the stabilization energy (ΔE_iso_) has been used [24] as a simple method to predict the efficacy of these antioxidants. In general, a decrease in the BDH_CH_ results in a better antioxidant activity. In Table 1, the computed BDH_CH_ and ΔE_iso_ values for C18-EdV and EdV have been reported, showing that the two derivative exhibit very similar values, with C18-EdV probably only slightly less effective than EdV. Furthermore, these values are in line with other similar compounds [24,32,33].

### 3.4. Protection against Free Radical-Mediated Lipid Peroxidation

The lipid peroxidation is responsible for damage and functional impairment of biological membranes; therefore, attention is given to the development of drugs able to suppress such oxidation. The antioxidant activity of EdV and the synthesized C18-EdV against radical-induced lipid peroxidation was evaluated in liposomes made up of egg-PC (PC, phosphatidylcholine), by measuring the percentage inhibition of aldehydic breakdown products (TBARS) produced during lipid peroxidation, using the TBA reactive. Either the water-soluble AAPH or the lipid-soluble AMVN was used as radical initiators in the aqueous phase or inside the lipid bilayer, respectively. The thermolysis of these azo-compounds produces tertiary carbon-centered radicals, which couple very rapidly with molecular oxygen generating a constant flux of peroxyl radicals, responsible for inducing lipid peroxidation by hydrogen abstraction upon the substrate. As shown in Figure 2, both the studied derivatives were able to inhibit lipid peroxidation although with a different extent when AMVN (Figure 2B) or AAPH (Figure 2C) is used. In more detail, in the presence of the water-soluble azo-initiator, the edaravone showed the best performance (percentage inhibition, 82%) since the free radical is produced initially in the aqueous phase, this result suggests that EdV is not completely encapsulated, and the free molecules can react with peroxyl radicals in the aqueous phase before the lipid peroxidation is started. Figure 2C shows instead the results obtained when the lipid-soluble initiator (AMVN) was used with PC liposomes. In this system, where the oxidation-initiating radicals are formed in the lipid phase, the C18-EdV exhibits the best antioxidant activities. Summarizing, these results suggest that the lipophilic group present in C18-EdV could be responsible for its greater localization in the bilayer with respect to edaravone; this could also explain its enhanced lipid peroxidation-inhibitory activity when the oxidation is induced in the lipid phase.

### 3.5. Affinity of EdV and C18-EdV for Lipid Bilayer

**Figure 3:**
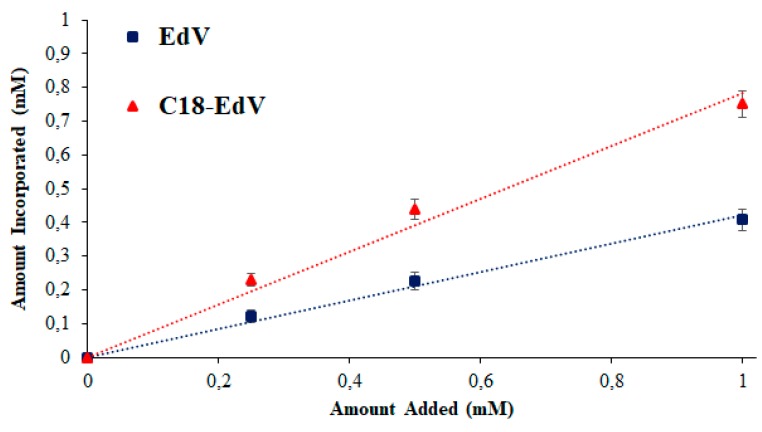
Amount of EdV and C18-EdV incorporated into liposomes with respect to their total amount. Increasing concentrations of EdV and C18-EdV in 0.1 mL of methanol were added to 0.9 mL of liposomal suspension. Their amount into liposomes was measured by the Folin-Ciocalteu method.

In order to better understand the role played by the lipophilic chain introduced in C18-EdV, the ability to interact with the lipid bilayer was evaluated for both antioxidants by measuring their percentage of incorporation in liposomes. Due to the structural similarity of the cell membrane with liposomes, they can be used as simple model systems to mimic biomembranes in a biologically more relevant way with respect to the octanol/buffer system. The amount of edaravone and C18-edaravone incorporated into PC lipid bilayer employed as cell membrane model are shown in Figure 3.

The amounts incorporated reflect the affinity of both antioxidants for the lipid bilayers of liposomes, and in principle for the cell membrane. In Figure 3, for both derivatives, a linear relationship can be observed, and it is possible to correlate their slopes with the affinity factors [34]. In these experiments, C18-EdV showed an affinity factor of 0.78 (R^2^ = 0.9919) which is about twice of EdV (0.42, R^2^ = 0.9919), likely thanks to the hydrocarbon chain added. As previously reported, [35,36] compounds with higher affinity can be incorporated more efficiently into the lipid bilayer of cell membranes. Upon these bases, the new derivative C18-EdV should be more efficient in protecting against free radicals-induced oxidative stress in cells.

### 3.6. Cellular Experiments

#### 3.6.1. Cytotoxicity in Human Retinal Epithelial Cells

**Figure 4:**
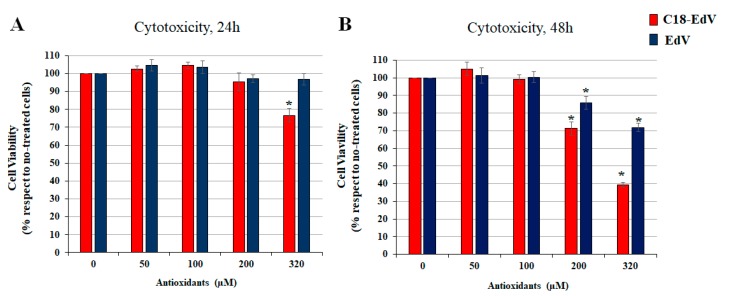
Cytotoxicity of C18-EdV in ARPE19 cells. The cells were treated with increasing concentrations of EdV, C18-edaravone for 24 h (**A**) and 48 h (**B**) in complete culture medium. The viability of treated-cells is reported as percentage respect with no-treated cells, which are considered as 100% of cell viability. The values in the figures are expressed as the means ± SD. Differences between control vs. C18-EdV or EdV were assessed using Tukey’s post hoc test, * *p* < 0.0001.

The toxicity of the newly synthesized C18-EdV was evaluated in ARPE-19 cells by MTT assay comparing it with that of edaravone (Figure 4). As shown in Figure 4, the treatment did not decrease cell viability after 24 h, while after 48 h a significative decrease of survival cells up to 100 µM was observed. From these results, it appears that the introduction of the C-18 lipid chain increases the cytotoxicity of edaravone, induced a decrease of viable cells both after 24 h and 48 h treatment. Although there are differences in the degree of cytotoxicity, both derivatives induced a marked cell death after 48 h of treatment in the presence of concentrations up to 100 µM (*p* < 0.0001). For this reason, we have chosen the 50 and 100 μM concentration of bioactive molecules for the experiments described in the next section.

#### 3.6.2. Enhancement of Edaravone Protective Efficacy by C18-Lipid Chain Introduction

**Figure 5:**
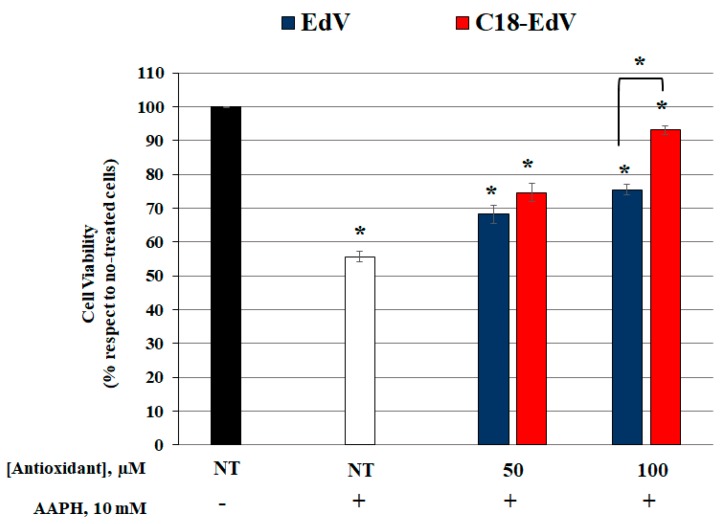
Effect of C18-Edravone in ARPE19 cells after AAPH exposure. The cells were pre-treated with 50 and 100 µM concentrations of C18-edaravone and edaravone for 24 h, before being exposed to 10 mm AAPH for 24 h. Cell viability was determined by MTT assay. Data are expressed as means ± S.D. of five independent experiments, each performed in triplicate. Differences between AAPH-treated cells vs. no-treated cells or EdV and C18-EdV treatments and between EdV vs. C18-EdV at 100 µM concentration were assessed using Tukey’s post hoc test, * *p* < 0.0001.

In order to better understand the C18-EdV potential ability to protect ARPE-19 cells from physiological stressors, oxidative damage mediated by AAPH was induced [37,38], comparing the C18-EdV activity with that of EdV. After pre-treatment of ARPE-19 with increasing concentrations of both antioxidants for 24 h, cells were washed twice with PBS to avoid direct extracellular interactions between the tested compounds and the oxidant. This step ensures that only the molecules that enter the cells, or that strongly interact with the lipid bilayer, may protect them from oxidative stress-induced death. The following incubation with 10 mm AAPH for 24 h gave the results shown in Figure 5.

In accordance with other studies, which demonstrated multiple roles of edaravone on the protection of retinal cells against oxidative stress [9,39,40], we demonstrated that EdV administration effectively decreases the oxidative stress-induced cell death in ARPE19 cells (~18% more cell viability than AAPH-exposed cells, *p* < 0.0001), at a concentration of 100 µM or less. However, C18-EdV was more efficient than EdV in contrasting the cellular oxidative damage induced by AAPH. The treatment with C18-EdV, at 100 µM concentration, induces an increase in cells survival of about 38% compared to AAPH-exposed cells (*p* < 0.0001) and up to 15% when compared to the unmodified molecule (*p* < 0.0001). The results shown here represent further evidence that the affinity of a molecule for the lipid bilayer can influence its biological activity. The different efficacy in AAPH-protection of the two antioxidants found in vitro with cultured ARPE19 cells reflects the amount incorporated during incubation. Therefore, the superior performance of C18-EdV respect to EdV can be ascribed to its ability to insert the hydrophobic C18 chain into the cell membrane bilayer; since part of the more hydrophilic EdV lies in the water phase, it will be brought away with the PBS washing step thus the antioxidant protection of retinal cells results inferior.

## 4. Conclusions

With the aim to improve the antioxidant activity of edaravone in living systems, specifically in lipophilic compartments, a C18 hydrocarbon chain was bonded at its pyrazolone ring at C-4. Such a strategy, leading to the synthesis of C18-EdV, did not produce changes in the radical scavenging activity, but the protection of lipid peroxidation was instead significantly improved compared to EdV. In more detail, the presence of lipophilic C18 chain increases the bilayer protection when the initiating radicals are generated in the lipid phase using AMVN, but a decrease is recorded when produced in the aqueous phase by means of the water-soluble initiator AAPH. This finding has been explained in terms of a more efficient insertion on the bilayer lipid matrix of C18-EdV with respect to EdV. For the same reason, the strong interaction occurring between the hydrophobic carbon chain of the lipidic tails of membranes with C18-EdV brings an increased affinity toward the cellular membrane producing an increased ability to protect retinal cells.

These data suggest that C18-EdV may work as a good antioxidant in cellular systems as well as in cell-free systems, resulting in promise for use as a novel therapeutic drug candidate in the treatment of diseases associate with oxidative stress. Further studies are in progress in our research group looking for a possible prediction of the structure and stability of the C18-EdV liposomes by means of computational protocols already assessed [41,42,43,44,45,46,47], in order to design, at a molecular level, efficient antioxidant nanovectors.

## Figures and Tables

**Table 1:** Calculated (B3LYP/6-311++G (d, p)) bond dissociation enthalpy (BDH) for the C-H bond at position 4 in a vacuum (1) and in methanol (PCM) (2).

Compound	BDH_CH_ (1)	ΔE_iso_ (1)	BDE_CH_ (2)	ΔE_iso_ (2)
EdV	82.36 kcal/mol	0	84.4 kcal/mol	0
	3.57 eV		3.66 eV	
C18-EdV	83.90 kcal/mol	1.17 kcal/mol	85.34 kcal/mol	0.90 kcal/mol
	3.64 eV	0.050 eV	3.70 eV	0.039 eV
